# Investigating the relationship between media multitasking and executive function within a military population

**DOI:** 10.1186/s41235-025-00634-5

**Published:** 2025-05-28

**Authors:** Scott Marriner, Julie Cantelon, Wade R. Elmore, Seth Elkin-Frankston, Nathan Ward

**Affiliations:** 1 United States Military Academy, West Point, NY USA; 2 Tufts University, Medford, MA USA; 3 US Army DEVCOM Soldier Center, Natick, MA USA

**Keywords:** Media multitasking, Cognitive performance, Executive function, Self-regulation

## Abstract

The pervasive nature of media multitasking in the last fifteen years has sparked extensive research, revealing a nuanced but predominantly negative association with executive function. Given the cognitive demands and technological landscape of the modern battlefield, there is a critical interest in understanding how these findings may or may not extend to military members. To understand this relationship, we investigated the hypothesis that self-reported media multitasking behaviors would be negatively associated with performance-based measurements of executive function in a military population. Results found no significant relationship between overall media multitasking and any measures of executive function. However, average media multitaskers performed significantly better than heavy media multitaskers in a task-switching paradigm. Furthermore, we examined whether self-regulation moderated this relationship. Unlike previous research in non-military samples, we did not find that the impact of media multitasking on executive function was more pronounced among military members with lower self-regulation. By uncovering the nuanced interplay between these variables, this research contributes to a more thorough understanding of the cognitive implications of media multitasking both in general and within a military context.

## Introduction

Society’s relationship with digital media is significantly changing as the speed, mobility, and affordability of digital media evolves. Digital media that originally included computing and television has evolved into mobile-based streaming, gaming, messaging, and more. These changes impact how media is consumed. One common form of media consumption, media multitasking, has attracted significant attention as society’s relationship with digital media changes.

Media multitasking is defined as the simultaneous performance of multiple activities including at least one form of media (Lang & Chrzan, 2015). This term has been applied broadly over the course of its study. Early examinations focused on the use of at least one media or technology type during multitasking with a non-media task (Ophir et al., 2009) while newer research tends to characterize the behavior as a balancing of multiple media or digital streams concurrently (Uncapher & Wagner, 2018). The changes in media multitasking characterization reflect the changes in its current societal prevalence. One study found that digital technology users commonly use multiple digital screens concurrently (Segijn et al., 2017). This behavior is supported in another study that reveals that over a third of online study participants engage in media multitasking while participating in online research (Drody et al., 2023).

As the prevalence of media multitasking has increased, the measurement of the behavior has also evolved. The original standard for measurement was the media multitasking index (MMI), a self-reported survey developed by Ophir and colleagues (2009). This exhaustive index assesses the simultaneous use of a combination of 12 different media categories during an average hour. These media categories include print media, TV, video on a computer, music, nonmusical audio, video or computer games, phone, instant message (IM)/chat, text messaging, email, reading on a computer, and other computer applications. Shortened versions of the MMI and other self-reported measures have also been developed to examine frequency-based instances of media multitasking (Baumgartner et al., 2017; Cain & Mitroff, 2011; Rioja et al., 2023).

Examining media multitasking is critical based on its links to performance across a variety of contexts. Increased media consumption has been associated with greater media multitasking and decreased academic performance in multiple student populations (May & Elder, 2018; Uzun & Kilis, 2019). This decreased academic performance includes poor learning behavior, lower grades, and poorer testing (Beuckels et al., 2021). Early reviews of media multitasking behavior in youth populations also indicated poorer emotional functioning and sleep quality among heavy media multitaskers (van der Schuur et al., 2015).

Most importantly, media multitasking has been implicated in poorer cognitive performance. The seminal study in this domain, conducted by Ophir and colleagues (2009), found that heavy media multitaskers performed worse than light media multitaskers in filtering out irrelevant information in memory and on task-switching tasks. A more nuanced picture has developed since then as some studies have failed to replicate these findings (Alzahabi & Becker, 2013; Wiradhany & Nieuwenstein, 2017). To add to the heterogeneity, recent reviews and meta-analyses have reported mixed relationships between media multitasking and cognitive performance across varying cognitive domains. For example, Kong and colleagues (2023) conducted a meta-analysis demonstrating that heavy media multitaskers significantly underperformed on all cognitive measures assessed compared to light media multitaskers. Parry and le Roux (2021) also found a negative association between media multitasking and cognitive performance though they interpreted the association as negligible given the small effect size. Uncapher and Wagner (2018) found that only certain components of cognitive performance had convergent, negative associations with media multitasking while others demonstrated a null relationship. The diversity of findings supports the need for continued examination.

### Media multitasking in a military context

Media multitasking has been examined in a variety of populations, yet the use of media multitasking in military populations remains unexplored. Most soldiers are young adults, a well-studied population, but few, if any, studies examine these behaviors in a military context. This is critical as media multitasking behavior permeates distinct workplaces and contexts. The US Army, and military more broadly, incorporates digital technology across many work tasks in ways that either require or encourage media multitasking. Digital technology is incorporated over other modalities in routine readiness aspects of military life like behavioral health screening (Bush et al., 2013). These changes in device use in the task saturated military environment further encourage media multitasking among service members. For example, new innovations in mixed-reality technology, like Microsoft Hololens, are used to enhance soldiers’ warfighting ability (Microsoft, 2021). These new technological innovations, meant to augment the soldier on the battlefield of the future, inherently force soldiers to media multitask. Therefore, the potential effects of media multitasking behavior on cognitive performance warrant examination with a particular focus on executive function.

### Media multitasking and executive function

Executive function describes the top-down cognitive mechanisms that allow for the selection and maintenance of attention in goal-directed behavior (Diamond, 2013; Miller & Cohen, 2001). This framework of executive function is comprised of three major components: inhibitory control, working memory, and cognitive flexibility (Diamond, 2013), each of which works in tandem to direct filtering, focusing, and switching underpinning attention. Examining the components individually provides greater clarity for their potentially unique relationship with media multitasking.

#### Associations between media multitasking and inhibitory control

Media multitasking has a mixed pattern of associations with inhibitory control performance. In a recent meta-analysis, Kong and colleagues (2023) concluded that heavy media multitaskers performed worse than light media multitaskers in tasks related to inhibitory control. Multiple meta-analyses also found small, but significant associations between media multitasking and increased distractibility within inhibition paradigms (Wiradhany & Koerts, 2021; Wiradhany & Nieuwenstein, 2017). However, non-significant associations have also been reported (Parry & le Roux, 2021; Uncapher & Wagner, 2018). These mixed findings may be attributed to the variety of measures used to assess inhibitory control.

Here, we focus on a widely used measure of inhibitory control, the Go / No-Go task. This task typically involves responding to target stimuli and refraining from responding to other stimuli. Multiple studies support a negative association between media multitasking and inhibitory control performance using a Go / No-Go task paradigm. For example, Gorman and Green (2016) found that heavy media multitaskers exhibited poorer performance on Go / No-Go task than light media multitaskers. Similarly, Shin and colleagues (2020) demonstrated that higher levels of media multitasking were associated with slower reaction times and more omission errors. Additionally, Murphy and Creux (2021) found that higher media multitasking scores were associated with poorer no-go trial accuracy in high task load conditions.

Several hypotheses have been proposed to explain the potential mechanisms underlying this negative relationship between media multitasking and Go / No-Go performance. First, Ophir and colleagues (2009) described heavy media multitaskers’ inability to filter irrelevant stimuli or information because of breadth biased cognitive control. In this description, more frequent media multitaskers scatter their attention more broadly across multiple streams of stimuli or sources of information. The introduction of multiple stimuli then leads to easy distraction from the primary task (van der Schuur et al., 2015). This hypothesis is known as the Scattered Attention Hypothesis. One rationale for attending to potentially task-irrelevant information is because that information may hold future use (Lin, 2009). Therefore, heavy media multitaskers may distribute attention across multiple sources of information to cast a wide net for potentially useful information. Regardless of purpose, people attending to multiple streams of information can exhibit decreased performance in tasks presenting both relevant information and irrelevant information.

In contrast, Uncapher and Wagner (2018) suggest that heavy media multitaskers may have a greater predisposition to exploratory behavior. This relationship would result in greater attentional lapses which affects goal-directed behavior. According to this logic, disruptive or frequent lapses in attention, as well as impulsive responses, lower the threshold for making decisions. This decreased inhibition leads to lower accuracy for task-irrelevant stimuli, and lapses in attention may also slow response rates.

Despite these prior findings and theoretical explanations, previous research has also failed to find a relationship between media multitasking and inhibitory control performance as measured by Go / No-Go tasks (Seddon et al., 2018). For example, Murphy and colleagues (2017) found no performance differences between light and heavy media multitaskers on a Go / No-Go task with mixed task loads. Of note, they found that average media multitaskers performed worse on that same task than the extreme groups. Ralph and colleagues (2015) also found no association between media multitasking level and cognitive performance on a modified sustained attention to response (SART) task that was similar to common Go / No-Go paradigms.

#### Associations between media multitasking and working memory

Unlike inhibitory control, negative associations between media multitasking and working memory have been demonstrated across a variety working memory measures. For example, overall, heavy media multitaskers performed worse than low media multitaskers on measures of working memory across multiple studies (Kong et al., 2023). Uncapher and Wagner (2018) suggested that this relationship may be driven by lower working memory performance in heavy media multitaskers. Multiple meta-analyses of cognitive performance in laboratory settings also point to a small but significant negative relationship between media multitasking and working memory (Parry & le Roux, 2021; Wiradhany & Nieuwenstein, 2017) though the effects vary depending on the specific measure of working memory used.

Here, we focus on the N-back task, which involves identifying if the current stimulus matches one from a specified number of trials prior (e.g., “Is this stimulus the same as the one from 2 trials ago?”). Despite the overall negative characterization between media multitasking and working memory, specific results with N-back tasks have been mixed. For example, studies have found that heavy media multitaskers performed worse, with increased false alarms and decreased accuracy, especially under high working memory load (Cain et al., 2016; Ophir et al., 2009; Ralph & Smilek, 2017). On the other hand, several studies found no relationship between media multitasking and N-back performance, contradicting earlier findings (Cardoso-Leite et al., 2016; Edwards & Shin, 2017; Luo et al., 2021; Wiradhany & Nieuwenstein, 2017).

#### Associations between media multitasking and cognitive flexibility

The association between media multitasking and cognitive flexibility is the most inconclusive of the executive functions based on mixed findings in both individual studies and meta-analyses (i.e., positive, negative, and null relationships) (Kong et al., 2023; Parry & le Roux, 2021; Uncapher & Wagner, 2018; Wiradhany & Koerts, 2021). This is in part due to the heterogeneity of tasks in cognitive flexibility paradigms.

For example, task-switching paradigms commonly show a negative relationship between cognitive flexibility and media multitasking. Ophir and colleagues (2009) found higher switch costs in heavy vs. light media multitaskers, with slower reaction times for both switch and repeat trials. A replication study by Wiradhany and Nieuwenstein (2017) showed increased switch costs in heavy multitaskers, but only for switch trials. Elbe and colleagues (2019) observed similar results, and Gorman and Green (2016) found lower accuracy in heavy multitaskers. These findings support the Scattered Attention Hypothesis, linking cognitive control to multitasking.

Conversely, task-switching performance has been positively associated with media multitasking. Alzahabi and Becker (2013) found that heavy media multitaskers had lower switch costs, performing better in task-switching scenarios. Alzahabi and colleagues (2017) also observed faster task-switching in heavier multitaskers. These results support the Trained Attention Hypothesis, which suggests that frequent media multitasking improves control processes involved in task-switching. Van der Schuur and colleagues (2015) propose that regular task-switching practice in heavy multitaskers may lead to training effects, enhancing cognitive flexibility, especially in laboratory-based executive function tasks. Therefore, training effects may transfer more readily and translate into improved performance for heavy media multitaskers in measures of cognitive flexibility that focus on task switching.

### Self-regulation as a moderating variable

Another important step in further characterizing the relationship between media multitasking and cognitive performance is examining potential moderating variables. One potential variable of interest given military contexts is self-regulation. Self-regulation is both a dynamic process and a choice problem that directs behavior to achieve desired outcomes and avoid undesired ones (Neal et al., 2017). Typically, people will monitor the output of their actions and measure it in relation to the desired goal. People will then face the choice of what to do (direction), when to do it, and how to do it (duration and intensity) to help fix the discrepancy between the current state of a variable and the desired goal.

Theoretically, executive functions can serve various functions for an individual’s ability to regulate goal pursuit, including during media multitasking. Hofmann and colleagues (2012) highlight how each individual component of executive function contributes during successful self-regulation in pursuit of a goal. For example, inhibitory control aids in the prevention of bad habits or impulses that do not directly contribute to goal completion. Likewise, working memory is critical to the accurate mental representation of the goal throughout the process and the prevention of attentional capture for tempting, irrelevant stimuli at the beginning of processing. Cognitive flexibility permits individuals to remain open to alternative courses of action during the process of goal completion. This flexibility can manifest itself as both a shift in means for successful goal completion or the temptation to shift the goal itself. These brief descriptions characterize the importance of the relationship between the individual components of executive function and successful self-regulatory behavior.

Several studies have also begun to explore this relationship between self-regulation, executive function, and media multitasking behavior. For instance, Parry and colleagues (2020) found that a self-regulation intervention created greater awareness among participants of their media multitasking behavior. This greater awareness increased instances in which they could disengage from multitasking and focus on a single task. Despite the increased awareness, the intervention did not produce the intended effect on executive function for this sample of civilians. Relatedly, Szumowska et al. (2018) found that self-regulation moderated the relationship between media multitasking and task switches in a voluntary task-switching paradigm. Specifically, high media multitasking was linked to more task switches in participants with low self-regulation. In a secondary study, Szumowska et al. (2018) also found that media multitasking negatively affected task performance in a free-switching condition, but this relationship did not hold in a forced-switching condition. These initial findings suggest that multitasking behavior can be altered through regulatory means; however, more research is needed to understand how these behavioral changes could impact cognitive performance, especially in military populations.

### Current study

Our primary objective is to characterize the relationship between media multitasking and cognitive performance in a novel, military population. To achieve this, we examined the relationship between the individual components of executive function and media multitasking with three key predictions. First, we predicted that media multitasking would be negatively correlated with inhibitory control as measured by a Go / No-Go task (Gorman & Green, 2016; Shin et al., 2020)—where higher levels of media multitasking will be associated with slower reaction times on go trials and the overall number of errors across all trials. Second, media multitasking will be negatively correlated with working memory as measured by an N-back task (Cain et al., 2016; Ophir et al., 2009)—where higher levels of media multitasking will be associated with lower accuracy on the 2-back trials. Finally, media multitasking will be negatively correlated with cognitive flexibility as measured by a task-switching task (Elbe et al., 2019; Ophir et al., 2009)—where higher levels of media multitasking will be associated with higher switch costs.

A secondary aim was to further characterize the relationship between media multitasking and cognitive performance by examining self-regulation as a potential moderating variable in a subset of the population. We predicted that high self-regulation would moderate the relationship between media multitasking and cognitive performance (Szumowska et al., 2018). Specifically, there would be a negative relationship between media multitasking and cognitive performance for those individuals who self-report lower in self-regulation.

## Methods

### Participants

One hundred and thirty-one active-duty US Army soldiers successfully participated in a larger study examining soldier tactical performance during a sustained live-fire exercise. Our sample was a convenience sample with no formal power calculations. Instead, the sample size was determined by the number of potential participants who could be recruited within the project timeline given our interest in this special population. After screening for outliers (detailed below in our Analysis section), one hundred and twenty-four soldiers (mean age ± SD = 23.14 ± 3.25; range 18 to 37 years) were included in the final analysis. Participants were recruited from a healthy soldier population within infantry units (Table 1).

**Table 1:** Sample demographic characteristics (*N* = 124 men, 0 women)

		Count
Education level	Some high school	1
	High school/GED	72
	Some college	33
	Associate’s degree	9
	Bachelor’s degree	9
Marital status	Single	80
	Married	41
	Divorced	3
Ethnicity	White	85
	Hispanic	19
	Black	6
	Asian	6
	Pacific Islander	4
	Other	3
	American Indian/Alaska native	1

Written informed consent was obtained from each participant. Recruitment included efforts to reduce the likelihood of command influence and peer pressure influencing participation. The US Army Combat Capabilities Development Command Armaments Center Institutional Review Board, Tufts University Institutional Review Board, and the Army Human Research Protections Office approved all procedures.

### Research design

A between-subjects design assessed the relationship between media multitasking and cognitive performance. Media multitasking was measured via self-report, and participants were grouped into light, average, and heavy media multitaskers based on self-reported levels. Cognitive performance was assessed by three tasks: a Go / No-Go task, an N-back task, and a task-switching task. Self-regulation was also measured via self-report for a subset of participants. Task descriptions, data preparation, and data analysis methods are described further below.

### Measures

#### Media multitasking index—short

Media multitasking was measured by the Media Multitasking Index- Short (MMI-S) developed by Baumgartner et al. (2017). This derivative of the original MMI assesses only 9 items primarily focused on simultaneous media behavior while watching television, social networking, and messaging. These measures were determined based on the elimination of media multitasking combinations with low prevalence in adolescents. Participants rate each item on a scale from 1 (“never”) to 4 (“very often”) based on their engagement with the media types. The items are then averaged to create an index of media multitasking frequency.

#### Short form self-regulation questionnaire

Self-regulation was measured through the Short Form Self-Regulation Questionnaire (SSRQ). The SSRQ is a 31-item questionnaire developed by Carey et al. (2004) based on the longer Self-Regulation Questionnaire developed by Brown, Miller, and Lawendowski (1998). The SSRQ was developed using a young adult population. Items are scored on a scale from 1 (strongly disagree) to 5 (strongly agree) based on a participant’s level of agreement. Some items are reversed scored. The outcome of the SSRQ is reported as a sum.

#### Executive function tasks

All three executive function tasks were completed on iPads and were based on versions from prior research with accuracy and response time as primary dependent measures of interest (Lee et al., 2012). Inhibitory control was measured with a Go / No-Go task. The Go / No-Go task required participants to respond with a button press for go conditions, a happy face, or withhold a response for no-go conditions, a sad face. The go condition was displayed 80% of the time compared to 20% for the no-go condition. Participants completed 10 practice trials followed by 50 test trials.

Working memory was measured with an N-Back test. Participants viewed a stream of numbers in the middle of the screen. Participants were tasked to indicate if the number on the current trial matched the trial directly before it (1-back) or two trials before it (2-back). Overall, participants completed 12 practice trials and 20 test trials.

Cognitive flexibility was measured through a task-switching task. The task-switching task presented participants with a number (1–4, 6–9) within a blue or pink box in the middle of the screen. If the box was pink, participants reported as quickly as possible with one hand whether the number was odd or even. If the box was blue, participants reported as quickly as possible with the other hand if the letter was higher or lower than 5. The color of the square could change between any trial. Participants completed three practice blocks of 12 trials each: one “low/high” task, one “odd/even task,” and one task-switch block. Participants then completed 48 trials of task-switching within the test block. In addition to accuracy and response time, we calculated switch cost by subtracting non-switch RTs from switch RTs.

### Procedure

The larger study, entitled “Characterizing Tactical Performance during Sustained Live-Fire Exercises,” utilized three phases to assess soldier performance. After providing informed, written consent, soldiers started phase 1 which included pre-mission baseline testing. The first part of pre-mission testing began at each soldier’s home station (Fort Campbell or Schofield Barracks) approximately one month prior to the field exercise. Soldiers completed a demographic questionnaire via Qualtrics. Soldiers then completed baseline measurements for a twelve cognitive task battery from BrainBaseline (Lee et al., 2012) on iPads.

The second part of pre-mission baseline testing occurred one day prior to the start of the field exercise training. Soldiers completed this part of testing at Fort Devens, Massachusetts. Soldiers completed the MMI-S via an iPad-administered Qualtrics survey in testing conditions like above. The first round of soldiers also completed the SSRQ using those same methods; however, we were unable to obtain SSRQ data for subsequent participants. Soldiers then completed phase 2 training which involved a battery of tests and tactical training over a 72-h field exercise. Lastly, the soldiers completed phase 3 which involved a recovery period from the field exercise. The recovery period also incorporated a variety of performance tests that are not currently included in the current report.

### Analysis

#### Correlational analysis

To prepare for analysis, we removed individual trials with non-plausible response times (i.e., RTs < 150 ms) from all cognitive task results. We did this because of prior recommendations for the minimum amount of time needed to perceive a stimulus and execute a motor response (Luce, 1986; Whelan, 2008). This resulted in the removal of 45 trials from the 6550 total Go / No-Go trials (0.7%), the removal of 6 trials from the 4810 total N-back trials (0.1%), and the removal of 285 trials from the 6110 total task-switching trials (4.7%). Next, we removed seven participants based on accuracy scores under fifty percent for either the Go / No-Go task or task-switching task. This resulted in a final sample of 124 participants for our primary analysis and 59 for our secondary analysis.

To assess the relationship between media multitasking and cognitive performance, we conducted bivariate correlations between MMI-S scores and measures for the individual components of executive function. These analyses first treated MMI-S scores as a continuous variable rather than using the common extreme groups approach to analyzing media multitasking. For inhibitory control, we conducted bivariate correlations between MMI-S scores and both accuracy and response time in Go trials. For working memory, we conducted a bivariate correlation between MMI-S scores and N-back accuracy in 2-back task loads. For cognitive flexibility, we conducted a bivariate correlation between MMI-S scores and RT switch cost in the task-switching paradigm. All bivariate correlations were two-sided. We used Pearson’s *r* for correlations except in cases involving non-normal distributions in which case we used Spearman’s Rho.

#### Moderation analysis

**Fig. 1:**
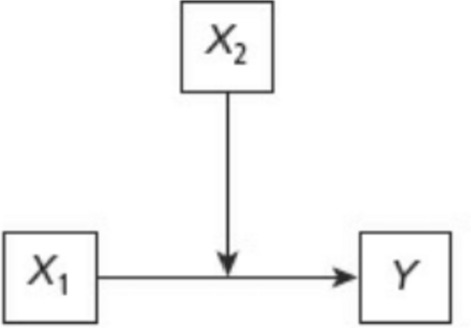
Basic Moderating Variable Model. X_1_ = MMI-S; X_2_ = SSRQ; Y = EFs

To assess the potentially moderating effect of self-regulation, we conducted a moderation analysis using an analysis of variance (ANOVA). Since all variables of interest were continuous, we utilized multiple regression with a focus on the interaction term. Similar to Szumowska and colleagues (2018), we assigned MMI-S score as the independent variable and SSRQ score as the moderator (Fig. 1). A simple slope analysis calculated the effect of media multitasking on cognitive performance at low, medium, and high values (- 1 SD, mean, + 1 SD) of self-regulation scores.

#### Exploratory analysis

In addition to our primary analyses, we examined media multitasking with a modified groups approach. Dichotomizing a continuous variable can be problematic (Preacher et al., 2005), but we took this approach as part of our exploratory analysis in part because most previous literature examined heavy versus light media multitaskers based on deviation from the mean. For our analysis, we conducted both an extreme groups approach and full group approach. First, we split the participant population into three even groups based on their MMI-S scores. We then conducted a one-way between-groups ANOVA to determine if there was a statistically significant difference between the groups. We then analyzed the data from the extreme groups approach where we split the population into light versus heavy media multitaskers based on a score higher or lower than one standard deviation from the mean. We conducted a one-way between-groups ANOVA to test for any differences between the groups.

### Additional analysis procedures

All statistical analysis was completed using R Studio, R Version 4.3.1. The following packages were used for the analysis: tidyverse (Version 2.0.0), ggplot2 (Version 3.4.4), moments (Version 0.14.1), psych (Version 2.4.1), corrplot (Version 0.92), lmtest (Version 0.9–40), car (Version 3.1–2), and interactions (Version 1.1.5). First, we conducted reliability tests on the MMI-S and SSRQ results. Normality of the questionnaire and cognitive tasks data were assessed using Shapiro-Wilks tests. Lastly, we utilized Bonferonni corrections for any post hoc pairwise comparisons.

## Results

Both the MMI-S (α = 0.89) and SSRQ (α = 0.85) demonstrated good internal consistency from a test of reliability. Responses to the MMI-S and SSRQ both produced a relatively normal distribution. Performance on the task-switching task produced a switch cost distribution that was also relatively normal; however, performance data on all Go / No-Go measures and the N-back task were not normally distributed. See Table 2 for descriptive statistics.

**Table 2:** Descriptive statistics

Questionnaire/task	Mean (SD)	Min	Max	Skewness	Kurtosis	Shapiro–Wilk W	Shapiro–Wilk *p*
MMI-S (*N* = 124)	2.54 (0.69)	1.00	4.00	0.11	2.37	0.98	0.12
SSRQ (*N* = 59)	112.1 (12.3)	88.0	142.0	0.26	2.44	0.98	0.42
GNG mean RT (*N* = 124)	489 (76)	301	736	1.01	4.34	0.93	< 0.001
GNG accuracy (*N* = 124)	96.30 (4.78)	76.90	100.00	− 2.19	7.78	0.71	< 0.001
N-back accuracy (*N* = 124)	75.87 (17.17)	16.70	100.00	− 1.01	3.99	0.92	< 0.001
TS switch cost (*N* = 124)	178 (136)	− 103	508	0.19	2.62	0.99	0.22
TS accuracy (*N* = 124)	94.20 (8.36)	50.00	100.00	− 3.09	14.55	0.65	< 0.001

MMI-S = Media Multitasking Index, SSRQ = Short, Short Self-Regulation Questionnaire, GNG = Go/No-Go, RT = response time, TS = task switch

### Cognitive task performance

**Fig. 2:**
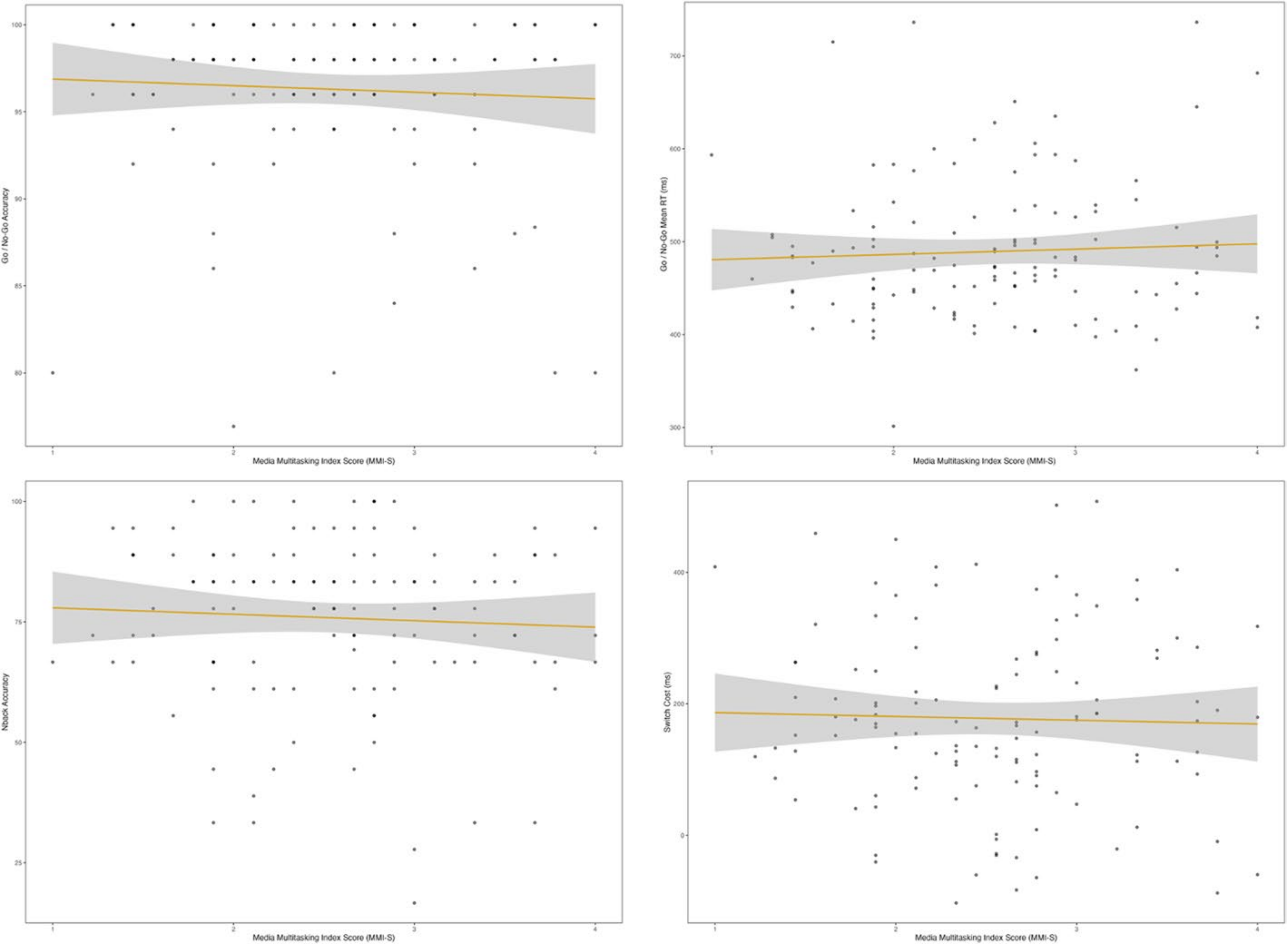
Scatterplots with linear trendlines between MMI-S and Go / No-Go Accuracy (top left), Go / No-Go Mean RT (top right), N-back Accuracy (bottom left), and Switch Costs (bottom right)

MMI-S scores were not significantly correlated with any of the cognitive task measures (Fig. 2; Table 3). Specifically, MMI-S scores were not significantly correlated with Go / No-Go accuracy (ρ(122) = − 0.02, *p* = 0.86, two-sided) or Go response times (ρ(122) = 0.03, *p* = 0.72, two-sided), nor with 2-back accuracy (ρ(122) = − 0.05, *p* = 0.57, two-sided). Similarly, MMI-S scores were not significantly correlated with switch costs (*r*(122) = − 0.03, *p* = 0.75, two-sided). Taken together, we did not observe a statistically significant relationship between media multitasking and executive function within our sample.

**Table 3:** Correlations

		MMI-S	GNG accuracy	GNG mean RT	N-back accuracy	TS switch cost
GNG accuracy	Pearson’s *r*	− 0.055				
	Spearman’s rho	− 0.016				
GNG mean RT	Pearson’s *r*	0.052	0.008			
	Spearman’s rho	0.033	0.044			
N-back accuracy	Pearson’s *r*	− 0.054	0.196*	− 0.145		
	Spearman’s rho	− 0.052	0.293***	− 0.136		
TS switch cost	Pearson’s *r*	− 0.029	− 0.026	0.024	0.082	
	Spearman’s rho	0.012	− 0.089	0.081	0.031	
SSRQ	Pearson’s *r*	− 0.108	− 0.04	− 0.116	− 0.068	− 0.043
	Spearman’s rho	− 0.093	− 0.163	− 0.075	0.004	− 0.081

MMI-S = Media Multitasking Index, SSRQ = Short, Short Self-Regulation Questionnaire, GNG = Go/No-Go, RT = response time, TS = task switch. *p < .05 ***p < .001

To determine the degree to which our data supported the null hypothesis (i.e., MMI-S was not associated with any of the EFs) or the alternative hypothesis (i.e., MMI-S was negatively associated with EF), Bayes Factors (BFs) supporting the alternative hypothesis (BF_10_) were computed using jamovi (v2.0). A BF for the alternative hypothesis indicates the degree to which the data is consistent with the alternative hypothesis, compared to the null hypothesis (Baniqued et al., 2015; Quintana & Williams, 2018). By convention, BF_10_ ≤ 0.33 provides moderate evidence in favor of the null hypothesis, while BF_10_ ≥ 3 provides moderate evidence in favor of the alternative hypothesis (Wagenmakers, Morey, & Lee, 2016). All BF_10_ values for the test of a relationship between MMI-S and EF provided at least moderate evidence in favor of the null over the alternative (all BF_10_ < 0.13).

### Moderation results

We conducted a multiple regression focused on the interaction of the independent variable, MMI-S scores, and the moderator, SSRQ scores. There was not a significant moderating interaction of self-regulation on media multitasking for any of the cognitive tasks. More specifically, the interaction between self-regulation and media multitasking was not significant for Go / No-Go accuracy (*b* = 0.19, SE = 0.10, *t* = 1.87, *p* = 0.07), and it was not significant for Go response time (*b* = 1.06, SE = 1.32, *t* = 0.80, *p* = 0.43). The interaction between self-regulation and media multitasking was also not significant for both 2-back accuracy (*b* = 0.19, SE = 0.30, *t* = 0.63, *p* = 0.53) and switch cost (*b *= − 3.23, SE = 2.66, *t* = − 1.22, *p* = 0.23).

### Exploratory analysis

We utilized two additional methods for analyzing the relationship between media multitasking and cognitive performance based on prior research. The first results compared light, average, and heavy media multitasking groups using analysis of variance (ANOVA). There was no significant effect of media multitasking group and cognitive task performance for Go / No-Go accuracy (*F*(2, 121) = 1.05, *p* = 0.35) or Go response time (*F*(2,121) = 0.11, *p* = 0.90). This lack of a significant group effect also held for 2-back accuracy (*F*(2,121) = 0.97, *p* = 0.38). Interestingly, there was a significant difference between media multitasking groups for switch costs (*F*(2, 121) = 4.78, *p* = 0.01). A Bonferroni post hoc analyses revealed this overall effect was driven by significant differences in switch costs (*p* = 0.01) between the average media multitasking group (M = 130 ms) and the heavy media multitasking group (*M* = 214 ms). There was no significant difference in switch costs between the light media multitasking group (*M* = 195 ms) and either of the average or heavy media multitasking groups (*p*s > 0.08).

The second group analysis included only extreme groups (light and heavy media multitaskers) based on standard deviation from the group mean on MMI-S. Independent two-sample *t*-tests were conducted to compare differences in cognitive performance between light and heavy media multitasking groups. There was no statistically significant difference between these extreme media multitasking groups for any of the executive function measures (*ps* > 0.16).

## Discussion

This study examined the relationship between media multitasking and cognitive performance in a novel, military population. We did not observe a significant relationship between media multitasking and executive function performance within this sample in terms of traditional NHST testing, and Bayes Factors all indicated moderate support in the direction of the null. These findings are in contrast with previous work demonstrating an overall small, but negative relationship between media multitasking and cognitive performance (Kong et al., 2023; Parry & le Roux, 2021), though not in contrast with other work that did not find relationships between media multitasking and executive function (Cardoso-Leite et al., 2016; Seddon et al., 2018).

Partitioning the sample into groups permitted a more nuanced examination of the relationship between media multitasking and cognitive task performance (but see Preacher et al., 2005 for risks associated with this approach). In our sample, heavy, intermediate, and light media multitaskers significantly differed only on task-switching performance. Surprisingly, the extreme groups approach, which is more commonly used, did not produce any statistically significant group effects for this sample. That said, our results are consistent with some previous research (Luo et al., 2021; Minear et al., 2013) that also did not find group differences in N-back and task-switching performance based on an extreme groups approach in a non-military context.

The lack of a significant relationship between media multitasking and inhibitory control was contrary to our predictions. Additionally, the small, but negative effect size characterized by previous meta-analyses was not observed. Rather our findings support the notion that media multitasking may not be related to inhibitory control within a military population, also supported by our post hoc Bayes Factor analysis. These results are consistent with previous findings that also did not observe a relationship between these variables in young adult populations (Murphy et al., 2017; Ralph et al., 2015; Seddon et al., 2018).

There was also no significant relationship between media multitasking and working memory. However, other studies utilizing different measures of working memory performance have also shown non-significant relationships with media multitasking (Edwards & Shin, 2017; Luo et al., 2021; Wiradhany & Nieuwenstein, 2017).

Like previous literature, the most divergent findings related to task-switching performance. Specifically, there was no significant relationship between media multitasking and task-switching when examining media multitasking as a continuous variable or as an extreme groups approach. Interestingly, there was a significant group effect when comparing media multitasking across light, average, and heavy users. Specifically, heavy media multitaskers displayed a significantly higher switch cost (worse task performance) than average media multitaskers. This finding is similar to previous research where heavy media multitaskers performed worse in task-switching (Elbe et al., 2019; Ophir et al., 2009); Wiradhany & Nieuwenstein, 2017); however, these studies used light media multitaskers as their comparison rather than average media multitaskers. In our study, although light media multitaskers had a smaller mean switch cost (better performance) than heavy media multitaskers, this numerical difference was not statistically significant.

Overall, there are several potential reasons that we did not observe a relationship between media multitasking and cognitive performance within this miliary sample. First, the young adults that comprise this sample may have developed differently and thus exhibit different behavior than young adults from earlier studies. As Uncapher and Wagner (2018) note, young adults today are being exposed to media at younger ages and therefore interact with it for longer periods of their developmental years. This exposure could influence both media multitasking behavior and its relationship to cognitive performance. It is worth noting that the military sample examined in this study were collectively heavier media multitaskers (MMI-S M = 2.54, SD = 0.69) than the original adolescent populations used to develop the short index (M = 2.22, SD = 0.77) (Baumgartner et al., 2017).

The context in which this sample engages with media may also affect their media multitasking behavior. Work contexts for soldiers are very different from home life and may restrict certain media use for significant time periods every week. Normal military work may also influence the types of media that a soldier typically engages with and how frequently they multitask. These work and context restrictions could potentially limit the effectiveness of the measures assessed here. These experiential factors could influence how the soldier cohort examined in this study engages with media multitasking and how that behavior then relates to cognitive performance.

The inclusion of self-regulation as a moderating variable provided a chance to further characterize the relationship between media multitasking and cognitive performance within this population. This analysis also did not find a significant relationship between media multitasking and cognitive performance when accounting for self-regulation scores. These results are consistent with previous literature (Parry et al., 2020; Szumowska et al., 2018) that also did not find a significant moderating effect of self-regulation on performance; however, in our study, we also had missing data for many participants on the self-regulation measure due to the nature of our study design and special population. This is one limitation of our study, and we highlight some additional limitations below as opportunities for future research.

## Limitations

Self-report measurement of media multitasking may have limited the conclusions that can be drawn in the present study. Self-report measures require an accurate self-assessment from participants. Humans are prone to both under and overestimate their media use (Uncapher & Wagner, 2018). Other authors have also pointed out how the addictiveness of smartphones and compulsive media use resulting from that trait can obscure accurate judgments of media use (Seddon et al., 2018). Future research could include behavioral or observation-based measures of media multitasking (Poplawska et al., 2021; Rosen et al., 2013). The inclusion of objectives measures may help to provide converging evidence for media multitasking characterization. This recommendation also extends to measuring self-regulation via objective, behavioral measures.

Additionally, measuring media multitasking via the MMI-S specifically could also have impacted these outcomes. Previous criticisms of the long-form MMI include not adapting to a fast-changing media landscape (Rioja et al., 2023). Streaming, podcasting, and content creation are all newer types of media that younger soldiers may be involved in. Other forms of older media, like video games, were excluded during the creation of the MMI-S. The lack of capture for certain newer and older media types may significantly affect how well the MMI-S represents media multitasking overall or within this sample. Future studies could address this by including additional measures of trait media multitasking, as well as more situational measures of media multitasking (Kazakova et al., 2015).

Another limitation stems from our cognitive tasks. More specifically, we chose one task per executive function (i.e., one inhibition task, one updating task, and one cognitive flexibility task) in part because of prior work based on dominant models of EF (Diamond, 2013; Miyake et al., 2000). It is possible that we did not observe more robust relationships between these EF tasks and media multitasking because of some oddities of the tasks themselves. Furthermore, we used versions of these cognitive tasks that are short, which has the advantage of minimizing participant fatigue and attrition. These tasks are based on versions validated with large online samples (Lee et al., 2012), and they have been used in a number of other peer-reviewed publications (Clark et al., 2017; Ehlers et al., 2017; Page et al., 2023; Rubin et al., 2021; Ward et al., 2022). That said, it is possible that the truncated number of trials meant that we were not able to get a more stable estimate of true performance for this soldier population. To overcome this, future studies should consider including multiple tasks for each of the EF subprocesses, as well as longer versions if time permits.

Finally, in the present study, we only explored soldiers with an infantry background. These soldiers potentially interact with media and technology differently on a regular basis than soldiers in other combat support roles (e.g., intelligence, communications). The context in which soldiers work may also affect differences in scoring on the MMI-S as well. If training effects hold, as explained in the Trained Attention Hypothesis, then these soldiers may exhibit different behaviors or performances than a larger military population that more frequently engages with media as part of its job. Additionally, understanding a soldiers’ purpose for engaging in media multitasking remains relatively unexplored (Uncapher & Wagner, 2018). Future research should examine media multitasking across various combat and non-combat roles and how this may or may not relate to cognitive performance.

## Conclusion

This study aimed to characterize media multitasking’s relationship with executive function within a novel, military population. We did not find a significant relationship between media multitasking and cognitive performance within this specific population; however, heavy media multitaskers did perform worse than average media multitaskers on a task-switching task measuring cognitive flexibility. Future research utilizing objective measures of media multitasking, as well as examining the tendency and purpose behind media multitasking across various military roles, will help clarify the degree to which this behavior can be used to predict cognitive performance. This insight will be especially important for informing future equipment design and technology adoption in the modern battlefield.

## Data Availability

The datasets used in the current study are available online: https://osf.io/js4tb/. The current study was not preregistered.
